# The genome of the Tiger Milk mushroom, *Lignosus rhinocerotis*, provides insights into the genetic basis of its medicinal properties

**DOI:** 10.1186/1471-2164-15-635

**Published:** 2014-07-29

**Authors:** Hui-Yeng Y Yap, Yit-Heng Chooi, Mohd Firdaus-Raih, Shin-Yee Fung, Szu-Ting Ng, Chon-Seng Tan, Nget-Hong Tan

**Affiliations:** Department of Molecular Medicine, Faculty of Medicine, University of Malaya, 50603 Kuala Lumpur, Malaysia; Plant Sciences Division, Research School of Biology, The Australian National University, Canberra, 0200 Australia; School of Biosciences and Biotechnology, Faculty of Science and Technology and Institute of Systems Biology, Universiti Kebangsaan Malaysia, 43600 UKM Bangi, Malaysia; Ligno Biotech Sdn. Bhd., 43300 Balakong Jaya, Selangor Malaysia; Malaysian Agricultural Research and Development Institute (MARDI), 43400 Serdang, Selangor Malaysia

**Keywords:** *Lignosus rhinocerotis*, Genome, Phylogeny, Secondary metabolism, Carbohydrate-active enzymes, Cytochrome P450 superfamily

## Abstract

**Background:**

The sclerotium of *Lignosus rhinocerotis* (Cooke) Ryvarden or Tiger milk mushroom (Polyporales, Basidiomycota) is a valuable folk medicine for indigenous peoples in Southeast Asia. Despite the increasing interest in this ethnobotanical mushroom, very little is known about the molecular and genetic basis of its medicinal and nutraceutical properties.

**Results:**

The *de novo* assembled 34.3 Mb *L. rhinocerotis* genome encodes 10,742 putative genes with 84.30% of them having detectable sequence similarities to others available in public databases. Phylogenetic analysis revealed a close evolutionary relationship of *L. rhinocerotis* to *Ganoderma lucidum*, *Dichomitus squalens*, and *Trametes versicolor* in the core polyporoid clade. The *L. rhinocerotis* genome encodes a repertoire of enzymes engaged in carbohydrate and glycoconjugate metabolism, along with cytochrome P450s, putative bioactive proteins (lectins and fungal immunomodulatory proteins) and laccases. Other genes annotated include those encoding key enzymes for secondary metabolite biosynthesis, including those from polyketide, nonribosomal peptide, and triterpenoid pathways. Among them, the *L. rhinocerotis* genome is particularly enriched with sesquiterpenoid biosynthesis genes.

**Conclusions:**

The genome content of *L. rhinocerotis* provides insights into the genetic basis of its reported medicinal properties as well as serving as a platform to further characterize putative bioactive proteins and secondary metabolite pathway enzymes and as a reference for comparative genomics of polyporoid fungi.

**Electronic supplementary material:**

The online version of this article (doi:10.1186/1471-2164-15-635) contains supplementary material, which is available to authorized users.

## Background

**Figure 1:**
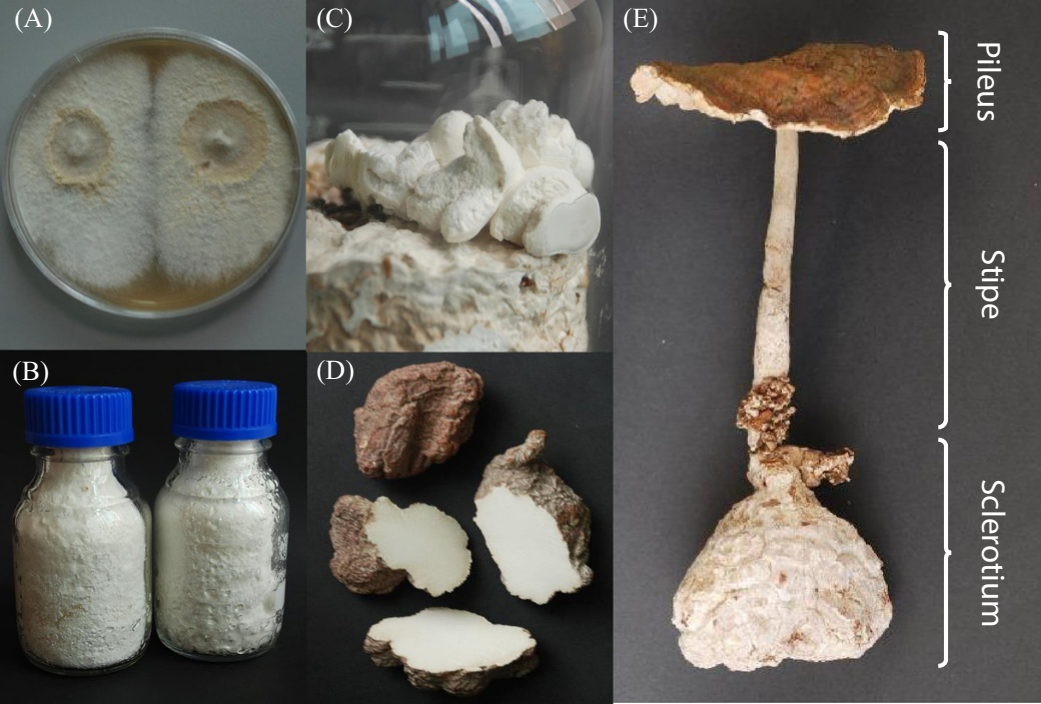
**Various stages of** ***L. rhinocerotis*** **development. (A)** Culture of *L. rhinocerotis* mycelium on nutrified agar, also known as the “Tiger-Eyes” (2 weeks culture). **(B)** Mycelial cultures of *L. rhinocerotis* on solid medium (1 to 2 months cultures). **(C)** Newly formed sclerotia on the surface of culture medium (4 to 6 months culture). **(D)** *L. rhinocerotis* sclerotia, the part with medicinal value. **(E)** Fruiting body of *L. rhinocerotis* with pileus (cap) and stipe (stalk) attached to the sclerotium.

*Lignosus rhinocerotis* (Cooke) Ryvarden, which belongs to the family of Polyporaceae, is characterized by a centrally stipitate pilei arising from its distinct tuber-like sclerotium. This mushroom is widely used by natives of Southeast Asia as a general health tonic for immune enhancement, or as a treatment regime for numerous ailments including cancer, asthma, and bronchitis. It is also used to treat discomfort caused by fright, fever, coughing, vomiting, and cuts [1]. The sclerotium is the part of *L. rhinocerotis* with medicinal value. It is a compact mass of hardened fungal mycelium and represents one of the stages in the fungal life cycle. This structure is a morphologically variable, nutrient-rich, multihyphal aggregate that serves as a food reserve and can remain dormant until favourable growth conditions arise [2]. Different developmental stages of *L. rhinocerotis* are shown in Figure 1. A two weeks culture of mycelial growth is shown in Figure 1A. Expansion of the mycelium by repeated branching of the germ tube (short, initial hypha) eventually develops into a circular form known as the “Tiger-Eyes”. Cross-linking of the radiating hyphae facilitates nutrient uptake and mobilization around the growing mycelium. After four to six months, the vigorous mycelial growth promotes the development of the sclerotium (Figure 1C). This is often spherical in shape with a dark and tough outer skin that keeps the internal compacted hyphal mass from drying out (Figure 1D). The stipe of the mushroom was formed after 12 months culturing, which was preceded by formation of the pileus (Figure 1E). In Malaysia, isolates of *L. rhinocerotis* have been found in Penang Island, Cameron Highlands, Hulu Langat, and Gerik. All of these isolates showed high nucleotide sequence identity (approximately 98%) in their internal transcribed spacer (ITS) gene regions [3, 4].

This mushroom is rich in carbohydrates and dietary fiber with moderate amounts of protein while being low in fat [5]. Previous research reported the medical benefits of *L. rhinocerotis* against hypertension, cancer cell cytotoxicity along with enhancement of immunomodulatory activity and antioxidant properties [5–8]. The non-digestible carbohydrates of *Polyporus rhinocerus*, a synonym for *L. rhinocerotis*, was also reported as a potential novel prebiotic for gastrointestinal health [9]. The recent interests in the nutrition and biopharmacology of *L. rhinocerotis* signalled for an immediate need to decipher its biochemical functions, at the genetic level and the identification of its bioactive components.

Rapid advancements in technology has led to the sequencing of numerous fungal genomes with the fungal kingdom becoming one of the most sequenced amongst the eukaryotes [10]. This is not unexpected due to their importance in industry, agriculture, medical, and health. However, the publicly available genome sequences of macrofungi, especially medicinal mushrooms, are still relatively scarce compared to the plant pathogenic and wood-degrading basidiomycetes or to ascomyceteous microfungi. A recent example is the genome sequence of the medicinal mushroom *Ganoderma lucidum* (lingzhi) by Chen et al. and Liu et al. where the genes in the triterpene biosynthesis and wood degradation pathways were described [11, 12]. Other genomes of edible mushrooms include *Volvariella volvacea* (straw mushroom) by Bao et al. [13] and *Agaricus bisporus* (button mushroom) by Foulongne-Oriol et al. [14].

In this paper, we present the *de novo* draft genome sequence of *L. rhinocerotis* TM02 sclerotium. The recent availability of several genome sequences of polyporaceous fungi, especially from the JGI CSP Saprotrophic Agaricomycotina Project [15], has allowed us to gain insights into the *L. rhinocerotis* genome through comparative analyses. We have also surveyed its secondary metabolite production capabilities and identified putative genes that may be involved in the biosynthesis of bioactive proteins and polysaccharides. To our knowledge, this is the first detailed description of the genomic features of *L. rhinocerotis*, an ethnobotanical mushroom of Southeast Asia.

## Results and discussion

### Genome features

Genome sequencing of *L. rhinocerotis* sclerotium with more than 100× coverage produced a total of 6,187 Mb clean data which was further assembled into a 34.3 Mb draft genome (Table 1). This consisted of 1,338 scaffolds with N50 of 90,329 bp and 53.71% G + C content. Using K-mer (15-mer) analysis with an average insert size of 700 bp, these scaffolds were estimated to cover 70.58% of the whole genome, which has an expected size of 48.6 Mb (Additional file 1). The lower genome coverage can be attributed to the high repeat rate encountered in the assembly. The repeat rate of the number of contigs with lengths shorter than 100 bp and longer than 100 bp is 27.26% and 7.89%, respectively (26.44% of total number of contigs) (Additional file 1). Heterozygosity is unlikely to be the major contribution to the lower genome coverage based on the 15-mer depth analysis (Additional file 1) [16]. The use of fosmid libraries for longer paired end reads (e.g. 10, 20, and 40 kbp), beyond the 5000 bp inserts we have used, may be required to overcome the high repeat rate. Alternatively, the genome assembly can be improved in the future by construction of whole genome physical maps using an optical mapping system, as reported for *G. lucidum* [11]. Nonetheless, this draft genome allows a detailed analysis into the gene content, phylogeny, and metabolic pathways of *L. rhinocerotis*.

**Table 1:** **Features of** ***L. rhinocerotis***

Scaffold features	
Total number	1,338
Total length (bp)	34,316,739
N50 (bp)	90,329
N90 (bp)	14,636
Max length (bp)	596,193
Min length (bp)	1,002
Sequence GC content (%)	53.71
**Genome features**	
Genome assembly (Mb)	34.3
Number of protein-coding genes	10,742
Coding sequences/genome (%)	44.31
Average gene length (bp)	1843.48
Average coding sequence length (bp)	1414.33
Average exon length (nt)	234.30
Average intron length (nt)	85.21
Average number of exons per gene	6.04

### Repeat elements

Repeat elements of 74 diverse families make up about 4.01% or 1,374,638 bp of the assembled genome of *L. rhinocerotis*, where 1.97% of them are tandem repeat sequences and 2.03% are transposable elements (TEs). The tandem repeats (<4 kbp), not clearly associated with transposons, vary in copy number from 1.8 to 362.5. While among the retrotransposons, long terminal repeats (LTRs) and non-LTR retrotransposons (long and short interspersed nuclear elements) make up 1.26% and 0.55% of the genome, respectively. DNA transposons (Class II) comprised 0.24% of the genome. The DNA transposons elements were mainly categorized into three classes: Enhancer (En/spm), Tigger (TcMar), and Activator (hAT).

### Gene prediction

The total 10,742 predicted genes, 216 tRNAs, 17 snRNAs and a single rRNA together comprise 44.33% of the assembled genome. Gene density and the average size of protein coding genes are 5.42 genes/10 kb and 1,414.33 bp, respectively. Among the tRNAs, 13 are possible pseudogenes, four with undetermined anticodons and the remaining 199 anti-codon tRNAs correspond to the 20 common amino acid codons. About half of the tRNAs (109) are predicted to not contain an intron. The genome size of *L. rhinocerotis*, its average gene length, the proportion of repeat sequences, and the average number of exons and introns were comparable to the recently sequenced polyporaceous *G. lucidum* genome [12].

The *L. rhinocerotis* genome revealed a total of 1,686 predicted genes that encode for hypothetical proteins with no apparent homologs to currently available sequences. This is indicative of the uniqueness of *L. rhinocerotis*. On the other hand, up to 8986, 5883, 6669 and 8997 genes are homologous to known proteins in the NCBI nr, SwissProt, InterPro and TrEMBL databases, respectively. These homologous proteins represent 84.30% of the assembled genome.

**Figure 2:**
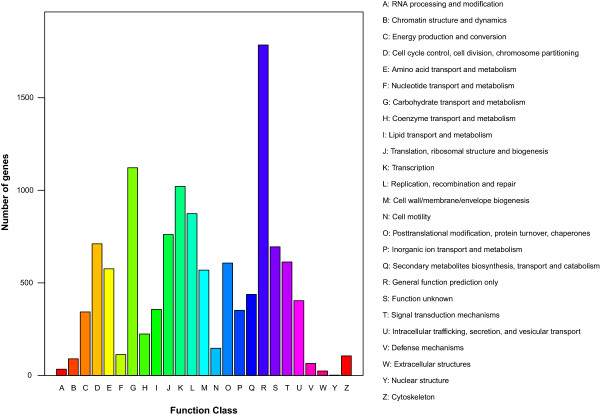
**KOG classification of proteins in** ***L. rhinocerotis*** **.** Distribution of predicted proteins from *L. rhinocerotis* genome according to functional class by Eukaryotic Clusters of Orthologs (KOG) database.

We further mapped the genome to the Eukaryotic Clusters of Orthologs (KOG), Gene Ontology (GO), and the Kyoto Encyclopedia of Genes and Genomes (KEGG) databases to further characterize the predicted proteins. Based on the phylogenetic classification of the KOGnitor, 4,948 proteins (46.06%) were assigned to KOG (Figure 2). The R category for “General functional prediction only” (typical prediction of biochemical activity), which has the most number of genes (1,785), indicates that these proteins were not unambiguously assigned to a certain group. This is followed by “Carbohydrate transport and metabolism”, “Transcription”, “Replication, recombination and repair”, and “Translation, ribosomal structure and biogenesis” as the top five most abundant gene categories in the KOG grouping. These findings suggest the presence of an enriched and varied array of carbohydrate uptake and metabolism mechanisms that can better facilitate the terrestrial substrate of *L. rhinocerotis* because such a lifestyle results in exposure to more diverse carbohydrate forms and sources as opposed to the wood saprophytes.

**Figure 3:**
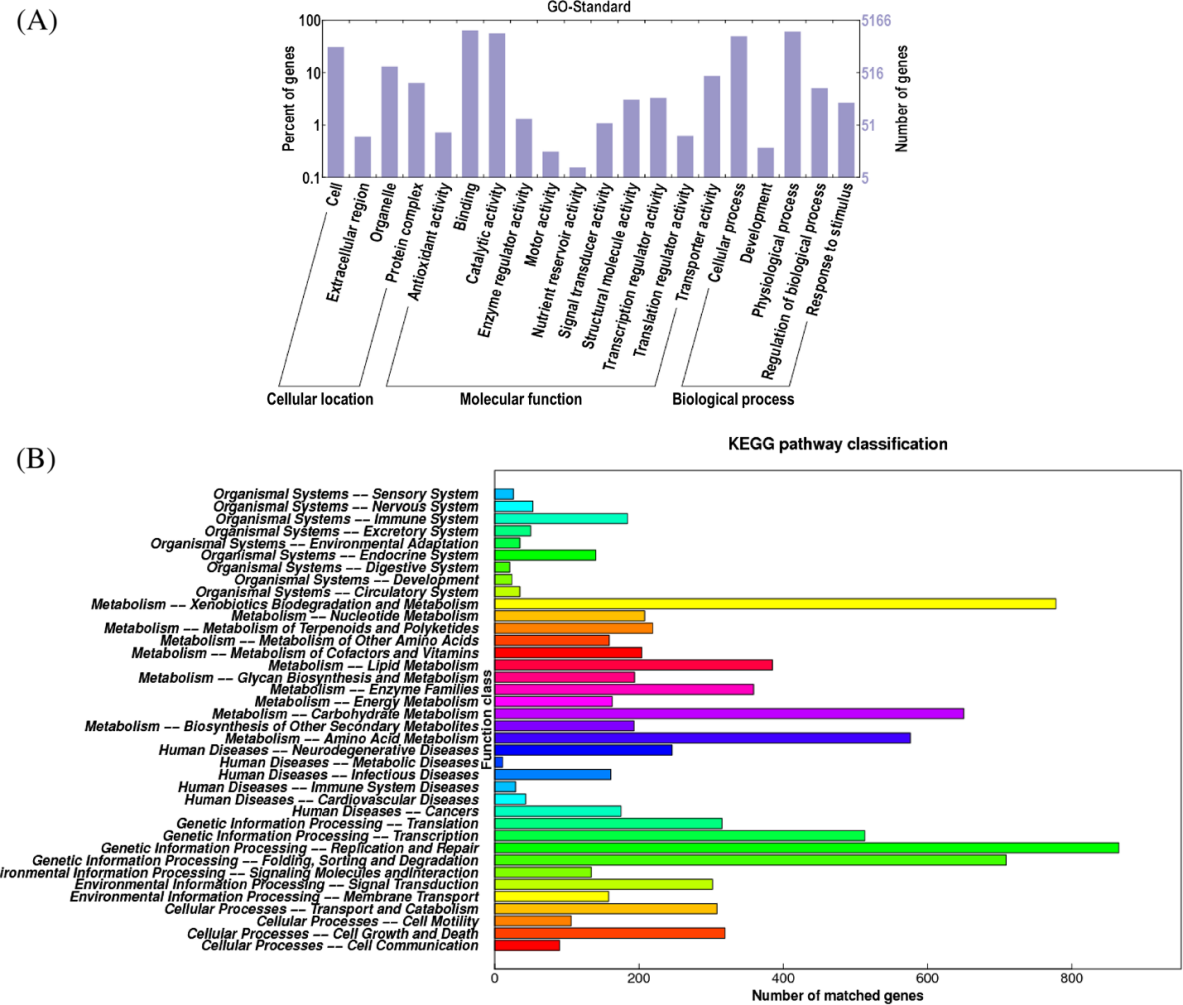
**GO and KEGG classifications of proteins in** ***L. rhinocerotis*** **.** Distribution of predicted proteins from *L. rhinocerotis* genome by **(A)** Gene Ontology (GO) and **(B)** Kyoto Encyclopedia of Genes and Genomes (KEGG) databases.

The hits from InterPro were used to map to the “Cellular location”, “Molecular function”, and “Biological process” categories of GO (Figure 3A). GO classification revealed that 49.28% (5,294) of the predicted proteins were mainly assigned to “Binding”, “Physiological process”, “Catalytic activity”, and “Cellular process”. To provide a better understanding of the gene functions in *L. rhinocerotis*, we successfully assigned 4,980 (46.36%) putative proteins to their orthologs in the KEGG database (Figure 3B). The top five categories in KEGG pathway classification, with the highest numbers of annotated genes in *L. rhinocerotis,* are “Genetic information processing - Replication and repair”, “Metabolism - Xenobiotics biodegradation and metabolism”, “Genetic information processing - Folding, sorting, and degradation”, “Metabolism - Carbohydrate metabolism”, and “Metabolism - Amino acid metabolism”.

### KEGG-based comparative genomics analysis

KEGG pathway annotations for seven medicinal Basidiomycota fungi, consisting of five agaricomycetes (*G. lucidum*, *Trametes versicolor*, *Wolfiporia cocos*, *A. bisporus*, *Pleurotus ostreatus*), a basidiomycete (*S. commune*) and tremellomycete (*Tremella mesenterica*), were selected for comprehensive comparison. *L. rhinocerotis* is relatively enriched with genes for catabolism and biosynthesis of secondary metabolites (Table 2, Additional file 2: Table S1) such as “Limonene and pinene degradation”, “Indole alkaloid biosynthesis”, “Penicillin and cephalosporin biosynthesis”, and “Stilbenoid, diarylheptanoid and gingerol biosynthesis”. However, *L. rhinocerotis* was predicted to have a relatively low number of genes associated with the biosynthesis of siderophore group nonribosomal peptides. The metabolism and biosynthesis of secondary metabolites in *L. rhinocerotis* are further discussed in the subsection “Secondary metabolism”.

**Table 2:** **Fungal genes distribution in P450 family and the third layer of KEGG pathways**

	L.rhi	G.luc	T.ver	W.coc	T.mes	S.com	P.ost	A.bis
**P450**	136	262	248	240	33	159	204	152
**KEGG pathway**								
00281: Geraniol degradation	17*	13	13	14	6	13	11	10
00900: Terpenoid backbone biosynthesis	13	14	14	17	14	20	14	18
00903: Limonene and pinene degradation	144*	79	72	75	29	53	64	56
01053: Biosynthesis of siderophore group nonribosomal peptides	2	110	119	82	97	141	64	53
00311: Penicillin and cephalosporin biosynthesis	7*	0	0	0	0	0	3	0
00312: beta-Lactam resistance	3*	0	0	0	0	0	0	0
00901: Indole alkaloid biosynthesis	4*	1	1	1	0	1	1	1
00945: Stilbenoid, diarylheptanoid and gingerol biosynthesis	83*	80	65	73	39	53	57	47
00960: Tropane, piperidine and pyridine alkaloid biosynthesis	32	26	46	19	8	20	24	18
00980: Metabolism of xenobiotics by cytochrome P450	33	113	146	119	12	95	91	90
00982: Drug metabolism - cytochrome P450	36	116	142	129	14	93	94	90

*represents *L. rhinocerotis* having relatively more genes than the average of seven Basidiomycota fungi. *Abbreviations*: L.rhi, *L. rhinocerotis;* G.luc, *G. lucidum;* T.ver, *T. versicolor;* W.coc, *W. cocos;* T.mes, *T. mesenterica;* S.com, *S. commune;* P.ost, *P. ostreatus;* A.bis, *A. bisporus*.

The Enzyme Commission (EC) number classification was used to link the respective enzyme genes to their repertoire of metabolic pathways [17]. In the fourth layer of the reference KEGG pathway, *L. rhinocerotis* was found to have 26 putative enzymes that are two-fold greater than the other fungi compared (Additional file 2: Table S2, Additional file 2: Table S3). Among them, 15 were mapped to “Metabolism”, two for “Genetic information processing” and one for “Organismal systems” while two remain “Unclassified” according to KO terms. There were another six enzymes that mapped to multiple sections in the first layer of KEGG.

Interestingly, a total of 535 enzymes are exclusive to *L. rhinocerotis* and not present in any other Basidiomycota fungi compared (Additional file 2: Table S4). Some of the exclusive enzymes participate in multiple KO classes. Among them, 79.25% are predicted to involve in “Metabolism” where 20.56% and 18.69% play major roles in “Amino acid metabolism” and “Carbohydrate metabolism”, respectively. This is followed by “Genetic information processing” (8.79%) and “Cellular processes” (5.61%). About 14.21% remain “Unclassified” without mapping to any specific pathway.

### Phylogeny of *L. rhinocerotis*

**Figure 4:**
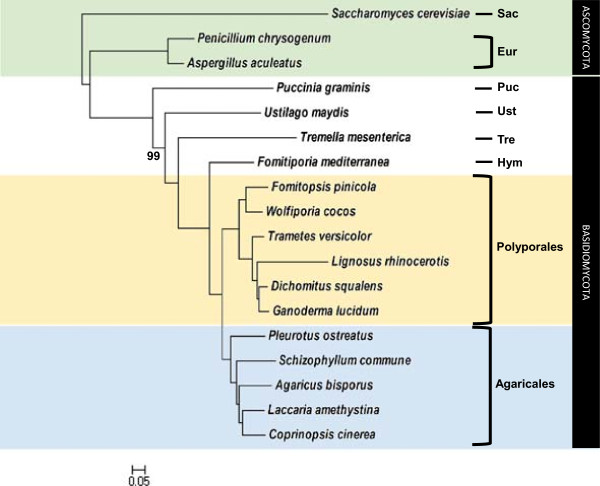
**Phylogenetic tree of** ***L. rhinocerotis*** **showing the evolutionary distance with different fungal species.** Phylogenetic tree construction from a concatenated alignment of 144 shared proteins rooting *Saccharomyces cerevisiae* as the outgroup. Branch lengths were estimated based on Bayesian inference and all bootstrap values are 100% unless otherwise specified on the lower left side of the internal node(s). Abbreviations: Sac, Saccharomycetales; Eur, Eurotiales; Puc, Pucciniales; Ust, Ustilaginales; Tre, Tremellales; Hym, Hymenochaetales.

In this study, we selected eight common KOGs shared by *L. rhinocerotis* and 14 Basidiomycota fungi plus three from Ascomycota fungi for rooting the phylogenetic trees. The four phylogenetic methods according to Kuramae et al. were employed [18]. The resulting single consensus tree gives highly supported internal branches of 99 to 100%, thus signifying the robustness of our dataset (Figure 4). Support value of 99% was only observed in the branch of *Puccinia graminis* to *Ustilago maydis* while the others are 100%. Phylogenetic analysis of the 144 concatenated proteins present in the 18 genomes compared revealed a closer evolutionary relationship of *L. rhinocerotis* to *G. lucidum*, *Dichomitus squalens*, and *T. versicolor*, all members of the Polyporaceae family. The Polyporales have been informally divided into several major clades, which include the antrodia, core polyporoid, and phlebiod clades that are well supported by a recent phylogenetic analysis [19]. *L. rhinocerotis* falls into the core polyporoid clade along with *G. lucidum*, *D. squalens*, and *T. versicolor* while *W. cocos* and *Fomitopsis pinicola* belongs to a sister clade known as the antrodia clade.

It should be noted that, although *L. rhinocerotis* falls into the same clade with *G. lucidum*, *D. squalens*, and *T. versicolor*, it is relatively distant from them and shows distinct morphological features. Unlike the other white-rot members from the core polyporoid clade that grow on wood, *L. rhinocerotis* has a terrestrial growth habit similar to the brown-rot *W. cocos* with the development of an underground sclerotium [20]. The sclerotium of *L. rhinocerotis* is oblong to irregular shape and its fruiting body (basidiocarp) is centrally stipitate with an isodiametric cap. On the other hand, the cap of *G. lucidum* and *T. versicolor* is offset and sometimes indistinct with either a bare stipe for the former or lacking one for both fungi. On the contrary, *D. squalens* has a basidiocarp with poroid hymenophore and lacks a stipe. Although *L. rhinocerotis* shows similar growth habit to *W. cocos* with the presence of a sclerotium, the latter has resupinate fruiting body and spherical sclerotium. Therefore, *L. rhinocerotis* is relatively unique among the sequenced Basidiomycota mushrooms.

### The CAZymes family

As *L. rhinocerotis* is known to thrive on cellulosic substrates, its genome was mapped to the CAZy database to identify the presence of carbohydrate-active enzymes (CAZymes) and auxiliary proteins [20]. A total of 332 non-overlapping CAZyme-coding gene homologs were identified. This includes 178 glycoside hydrolases (GH), 77 glycosyl transferases (GT), three polysaccharide lyases (PL), 102 carbohydrate esterases (CE), 205 carbohydrate-binding module (CBM), and 37 with auxiliary activities (AA) distributed among 39, 26, 1, 13, 32, and 6 coinciding EC activities respectively (Additional file 3). The mapped EC activities may not be directly associated with the family but simply a result of similarity to adjacent modules due to the modular nature of CAZymes. The number of CAZyme candidates identified was almost similar to the average reported in several studies for Basidiomycota fungi [12, 13]. The high number of putative GH and GT genes suggests their plausible roles in the degradation of plant cell wall polysaccharides. These polysaccharides consist mainly of cellulose, hemicellulose (including xylan, xyloglucan, glucogalactomannan, galactan, and respective side chains), and pectin (composed of galacturonan, rhamnogalacturonan, and respective side chains).

### The CYPs family

The cytochrome P450 (CYP) superfamily is a diverse group of enzymes involved in various physiological processes, including detoxification, degradation of xenobiotics and the biosynthesis of secondary metabolites [21]. Although not substantial, when compared to most other fungi, it is noted that *L. rhinocerotis* has 33 genes engaged in “Metabolism of xenobiotics by cytochrome P450” and 36 in “Drug metabolism - cytochrome P450” KEGG sub-pathways (Table 2), respectively.

For further comparison, the P450 genes in *L. rhinocerotis* and seven other basidiomycetes were retrieved using BLAST searches against the P450 database (Table 2). *G. lucidum* had the most number of putative P450 genes of 262 followed by *T. versicolor* (246 functional genes and two known pseudogenes) and *W. cocos* (238 functional genes and two known pseudogenes). On the other hand, *T. mesenterica*, a tremellomycete, formed the smallest group among the eight fungi compared with 32 functional genes and a known pseudogene. *L. rhinocerotis* had a total of 136 CYP sequences (135 functional genes and a known pseudogene), which can be classified into 37 families according to Nelson’s nomenclature (Table 2, Additional file 2: Table S5) [22]. The CYP5144 family was found to have the most number of genes (35 genes), followed by CYP5150 (20 genes) and CYP5037 (14 genes) families (Table 3). The CYP5144 family may play a role in triterpenoid biosynthesis (see subsection “Secondary metabolism”) while genes from the CYP5037 and CYP5065 families were found to cluster with terpene synthases (Additional file 2: Table S6). *L. rhinocerotis* also harbours five genes from the CYP63 family, which has been implicated in xenobiotic degradation in *Phanerochaete chrysosporium* [23]. However, the exact roles of these CYPs remain to be determined.

**Table 3:** **P450 genes and subfamilies in** ***L. rhinocerotis***

Family	Subfamily	Corresponding gene number	Total gene number
CYP5144	A; C; D; F	6; 23; 1; 5	35
CYP5150	A; B; C	17; 1; 2	20
CYP5037	A; B	2; 12	14
CYP504	E	6	6
CYP512	A	6	6
CYP5035	A; B; D	3; 2; 1	6
CYP63	A	5	5
CYP505	D; G	3; 2	5
CYP620	E	3	3
CYP5136	A	3	3
CYP5139	A	3	3
CYP66	A; B	1; 1	2
CYP5046	A	2	2
CYP5065	A	2	2
CYP5151	A; B	1; 1	2
Others	—	—	22

Others are minority CYP families with single gene number which are not shown here.

### Secondary metabolism

Secondary metabolite biosynthetic genes are often clustered [24]. The *L. rhinocerotis* genome contains several secondary metabolite gene clusters that suggest the potential for production of certain biologically active compounds (Additional file 2: Table S6). There are ten gene clusters encoding key enzymes, such as terpene synthases (TS), non-ribosomal peptide synthetase (NRPS), and polyketide synthase (PKS), that are crucial for the biosynthesis of terpenes, peptides, and polyketides, respectively. It is noted that, like most basidiomycetes, *L. rhinocerotis* has very few PKS genes and multi-domain NRPS genes compared to ascomycetes. The only PKS gene that can be found in *L. rhinocerotis* is GME5066_g, which encodes a non-reducing PKS which are often associated with the biosynthesis of aromatic polyketides. This non-reducing PKS appears to be conserved among basidiomycetes and an ortholog of the gene can be found in most of the sequenced basidiomycetes genomes, including *G. lucidum*, *T. versicolor*, and *A. bisporus*. Interestingly, GME5066_g shared a head-to-tail homology (38% identity and 55% similarity) and domain architecture with the orsellinic acid synthase from *Coprinopsis cinerea* (CC1G_05377), the only basidiomycete PKS gene that has been characterized so far [25]. Like CCIG_05377, GME5066_g contains a starter unit acyl-carrier protein transacylase (SAT), ketosynthase (KS), acyltransferase (AT), product template (PT), two acyl-carrier proteins (ACPs) and a thioesterase (TE) domain. GME5066_g is clustered with GME5065_g, which is a predicted flavin-dependent oxidoreductase. It remains to be determined if the GME5066_g gene cluster produces orsellinic acid derivatives or related polyketides. The *L. rhinocerotis* genome also harbours a single multidomain NRPS gene. The NRPS has a single adenylation domain along with three thiolation and condensation domains, and are conserved among several basidiomycetes, including *D. squalens* DICSQDRAFT_132068 (61% identity) and *T. versicolor* TRAVEDRAFT_27949 (59% identity), but none are characterized.

Terpenoids (or isoprenoids) is one group of secondary metabolites that are well recognized for their pharmaceutical uses and are known to be one of the major groups of therapeutic compounds in *G. lucidum*. The triterpenoid ganoderic acids, for example, have been reported to have anti-tumor, immuno-regulation, and antioxidative functions [26]. Other notable examples include the diterpenoid antibiotic pleuromutilins from *Clitopilus* mushrooms [27], as well as the sesquiterpenoid anticancer illudins [28]. In the genome of *L. rhinocerotis*, we identified 15 key enzymes involved in the mevalonate (MVA) pathway but not the 2-C-methyl-D-erythritol 4-phosphate/1-deoxy-D-xylulose 5-phosphate (MEP/DOXP) pathway (Additional file 4). This indicates that the terpenoid backbone biosynthesis in *L. rhinocerotis*, like most fungi, can only proceed via the MVA pathway. All the core enzymes involved in the MVA pathway are listed in Table 4. The enzymes 3-hydroxy-3-methylglutaryl-CoA reductase and solanesyl diphosphate synthase are each encoded by two copies of the genes whereas the remaining nine enzymes are encoded by single copy genes. As in the case for the *G. lucidum* genome reported by Liu et al. [12], we found a fusion gene (GME67_g) that was partially homologous to cystathionine beta-lyase (metC; K01760; EC4.4.1.8) and mevalonate kinase (MVK; K00869; EC2.7.1.36) at the N- and C-terminal respectively. This metC-MCK gene, which encodes for 918 amino acids, is the only gene that matches the key enzyme mevalonate kinase, implying the role in terpenoid backbone biosynthesis.

**Table 4:** **Putative genes involved in terpenoid backbone biosynthesis**

Gene name and definition	EC No.	KO term	Gene ID
E1.1.1.34; 3-hydroxy-3-methylglutaryl-CoA reductase	1.1.1.34	K00021	GME845_g;
			GME10429_g
metC; cystathionine beta-lyase (homologous to mevalonate kinase at the C-terminal)	4.4.1.8	K01760	GME67_g
atoB; acetyl-CoA C-acetyltransferase	2.3.1.9	K00626	GME4142_g
FDPS; farnesyl diphosphate synthase	2.5.1.1 2.5.1.10	K00787	GME3582_g
GGPS1; geranylgeranyl diphosphate synthase, type III	2.5.1.1 2.5.1.10 2.5.1.29	K00804	GME4885_g
mvaK2; phosphomevalonate kinase	2.7.4.2	K00938	GME148_g
MVD; diphosphomevalonate decarboxylase	4.1.1.33	K01597	GME2603_g
E2.3.3.10; hydroxymethylglutaryl-CoA synthase	2.3.3.10	K01641	GME2144_g
idi; isopentenyl-diphosphate delta-isomerase	5.3.3.2	K01823	GME7794_g
hexPS; hexaprenyl diphosphate synthase	2.5.1.33	K05355	GME1421_g
SPS; solanesyl diphosphate synthase	2.5.1.11	K05356	GME7467_g;
			GME8769_g
DHDDS; cis-prenyltransferase, dehydrodolichyl diphosphate synthase	2.5.1.-	K11778	GME2072_g

We next searched the *L. rhinocerotis* genome for potential triterpenoid biosynthesis genes and found an open reading frame (GME631_g) that encodes a single copy gene for lanosterol synthase (LSS; K01852; EC5.4.99.7). LSS is a squalene/oxidosqualene cyclase family enzyme that catalyzes the cyclization of the triterpenes squalene or 2-3-oxidosqualene to lanosterol, the precursor of all steroids [29]. This enzyme has been implicated in biosynthesis of ganoderic acids, which are the bioactive triterpenes in *G. lucidum* [11]. Similarly, the LSS in *L. rhinocerotis* can be involved in biosynthesis of bioactive triterpenes. The CYP5144 and CYP512 families of P450 genes have been previously implicated in triterpenoid biosynthesis in *G. lucidum* due to their co-expression with LSS [11]. As mentioned earlier, CYP5144 is the largest P450 family in *L. rhinocerotis* with 35 genes, while six CYP512 family genes are present in its genome as well. This suggests that *L. rhinocerotis* may be a potential triterpenoid source.

Mushrooms are known to be prolific producers of bioactive sesquiterpenes [30]. The *L. rhinocerotis* genome is enriched with sesquiterpenoid biosynthesis genes compared to some of the other seven Basidiomycota genomes (Additional file 2: Table S1). The *L. rhinocerotis* genome encodes up to 12 sesquiterpene cyclase genes. This is comparable to the number of sesquiterpene cyclase genes found in the recently sequenced genome of the Jack O’Lantern mushroom *Omphalotus olearius*, which is the producer of anticancer illudins [31]. Many of these terpene synthase genes are clustered together with various modifying enzymes (Additional file 2: Table S6). Comparatively, the common filamentous ascomycetes have fewer sesquiterpene cyclase genes, for example, both the *Aspergillus nidulans* and *Aspergillus oryzae* have only one sesquiterpene cyclase gene, while the *Aspergillus fumigatus* has none [32]. This suggests that unlike the filamentous ascomycetes that are enriched with the polyketide and nonribosomal peptide biosynthesis genes, the sesquiterpenoids are major secondary metabolites produced by *L. rhinocerotis*.

### Biosynthesis of potential bioactive proteins and polysaccharides

Polysaccharides are the most extensively investigated mushroom constituents due to their pharmaceutical potential. The water soluble 1,3-β- and 1,6-β-glucans are some of the most active immunomodulatory compounds reported [33]. Additional file 2: Table S7 lists enzymes that may be involved in the biosynthesis of uridine diphosphate glucose (UDP-glucose, precursor of glucans) and 1,6-β-glucans in *L. rhinocerotis*. These enzymes include hexokinase, α-phosphoglucomutase, UTP-glucose-1-phosphate uridylyltransferase, 1,3-β-glucan synthases, and β-glucan biosynthesis-associated proteins.

Mushrooms have also been an important source of bioactive proteins, which include lectins, fungal immunomodulatory proteins (FIP), ribosome inactivating proteins (RIP), antimicrobial proteins, ribonucleases, and laccases [34]. The genome of *L. rhinocerotis* codes for nine putative lectins, two putative fungal immunomodulatory proteins (FIP), and four putative laccases (Additional file 2: Table S7). It is interesting to note that both of the *L. rhinocerotis* FIPs (GME7566_g and GME10641_g) are homologous to LZ-8 (64% identities), a member of the FIP family from *G. lucidum* that has been shown to possess immunomodulation and anti-cancer activity [35, 36]. Both the putative FIP proteins carry an Fve domain (pfam09259) found in the major fruiting body protein isolated from *Flammulina velutipes* with immunomodulatory activity [37].

## Conclusions

*L. rhinocerotis* genome sequencing has allowed us to perform comparative genomics and phylogenetic analysis. This has provided valuable insights into the biology of this medicinal mushroom from Southeast Asia. A survey of the secondary metabolite biosynthesis genes in the *L. rhinocerotis* genome shows that it is particularly enriched with sesquiterpenoid biosynthesis genes. Thus, future bioactive molecule discovery efforts should focus on this class of metabolites. Furthermore, *L. rhinocerotis* appears to encode the capabilities to produce 1,3-β- and 1,6-β-glucans as well as bioactive proteins, such as lectins and FIPs. Our genomic analysis of *L. rhinocerotis* will provide the foundation for future research and exploitation of *L. rhinocerotis* in pharmacological and functional food applications.

## Methods

### Fungal material, sequencing, and assembly

Sclerotium of *L. rhinocerotis* was collected from tropical forest at Lata Iskandar, Cameron Highland, Pahang, Malaysia (4.3245°N, 101.3324°E) in 2010. The fungus was deposited at Royal Botanic Gardens, Kew (Richmond, London, England) with the accession number K(M) 177812. Genomic DNA was extracted using a modified cetrimonium bromide (CTAB) procedure [38] from the sclerotium of *L. rhinocerotis* TM02 strain cultivated by Ligno Biotech Sdn. Bhd. (Balakong Jaya, Selangor, Malaysia). Paired-end reads were generated by sequencing of four cloned insert libraries of 200, 700, 2,000, and 5,000 bp using Hiseq2000 system (Illumina Inc., San Diego, CA, USA) at BGI-Shenzhen, China. Reads of low complexity and low quality with adapter and duplication contamination were removed from the raw data to strengthen the accuracy of follow-up analysis. To avoid issues associated with heterozygosity and/or low sequence quality, reads with significant poly-A structure and kmer frequency of 1 were removed. The clean short reads were then assembled using SOAPdenovo based on *de Bruijn* graph theory [39]. The gaps were filled using the GapCloser module from SOAPdenovo. During scaffold construction, contigs with certain distance relationships but without genotypes were connected with wildcards. The GapCloser module then replaced these wildcards using the context and paired-end reads information. The GapCloser assembled sequences iteratively in the gaps to fill large gaps where at each iterative cycle, GapCloser considered only the reads that could be aligned in the current cycle.

### Gene prediction and annotation

Protein coding genes were predicted using the *ab initio* gene predictors Augustus [40], GeneMark-ES [41], and SNAP [42]. The resulting gene sets were integrated to get the most comprehensive and non-redundant reference gene. Transposon sequences were predicted by aligning the assembled gene sequences with the transposon Repbase database [43] using RepeatMasker software at http://www.repeatmasker.org and RepeatProteinMasker software (transposon protein library). Tandem repeat sequences were predicted using Tandem Repeat Finder (TRF) [44]. rRNA sequences were identified by rRNA pool alignment and rRNAmmer *de novo* prediction software [45]. tRNA genes were predicted by tRNAscan-SE software [46] while others non-coding RNAs (miRNA, sRNA, and snRNA) were predicted by Rfam.

Predicted genes were functionally annotated based on homology to annotated genes in various databases via BLAST. Protein models were aligned to SwissProt, TrEMBL, InterPro and NCBI nr (BLASTP cut-off e-value ≤ 1e-5); and further classified according to GO [47], KOG [48], and KEGG pathways [49]. KEGG terms are assigned into four layers. The first layer consists of seven main sections, including “Metabolism”, “Genetic information processing”, “Environmental information processing”, “Cellular processes”, “Organismal systems”, “Human diseases”, and “Drug development”; and is further divided into several small entries, the second layers. The third and fourth layers are the specific pathway map and specific genes regulated in each pathway, respectively.

### Phylogenetic analysis

Together with *L. rhinocerotis*, 14 Basidiomycota of agaricomycetes (*G. lucidum*, *T. versicolor*, *D. squalens*, *W. cocos*, *F. pinicola*, *Fomitiporia mediterranea*, *A. bisporus*, *Coprinus cinereus* (synonym *C. cinerea*), *Laccaria amethystina*, *P. ostreatus*), basidiomycete (*S. commune*), tremellomycete (*T. mesenterica*), pucciniomycete (*P. graminis*), and ustilaginomycete (*U. maydis*) were selected for analysis. Three Ascomycota of eurotiomycetes (*Aspergillus aculeatus*, *Penicillium chrysogenum*) and saccharomycete (*Saccharomyces cerevisiae*) were added to root the phylogenetic trees. All the selected genomic gene models were downloaded from the US Department of Energy Joint Genome Institute website at http://genome.jgi.doe.gov/ (Additional file 2: Table S8).

Protein sequences of each shared KOG family from the different species were aligned using CLUSTAL X [50]. The multiple sequence alignments were concatenated using DAMBE5 [51] upon the removal of poorly aligned regions by GBlocks server [52]. PROTTEST [53] was used to select the best fit empirical substitution model of protein evolution for the concatenated alignment.

Maximum Parsimony and Neighbor Joining analyses were executed with Phylip [54] using PROTPARS and PROTDIST (JTT model). TREE-PUZZLE [55] was used to construct maximum likelihood quartet puzzling trees using the VT model [56]. Bayesian inference using Markov chain Monte Carlo phylogenetic analysis was performed with MrBayes 3.2.1 [57] under Rtrev + G + F model. Trees were sampled every 10 generations over a random starting trees of 50,000 generations, resulting in a total of 5,000 sampling trees, of which the first 2,000 were discarded. The remaining 3,000 trees were used to build a single consensus tree with ≥ 70% majority-rule using MEGA [58].

### CYP and CAZy family classifications

*L. rhinocerotis* gene models were aligned to fungi P450 sequences and the detected CYPs were named according to nomenclature in the P450 database (cut-off e-value ≤ 1e-10, identity > 30%) at Cytochrome P450 homepage (http://drnelson.uthsc.edu/CytochromeP450.html) [22]. Annotation of carbohydrate-active enzymes in *L. rhinocerotis* genome was carried out by BLASTP analysis against CAZy database at http://www.cazy.org/ [59].

### Secondary metabolites gene clusters annotation

Secondary metabolite gene clusters were determined using Secondary Metabolite Unique Regions Finder (SMURF, http://jcvi.org/smurf/index.php) [60] based on PFAM and TIGRFAM resources along with the gene’s chromosomal position and antibiotics & Secondary Metabolite Analysis Shell (antiSMASH 2.0, http://antismash.secondarymetabolites.org/) [61]; a web-based analysis platform.

### Accession number

This Whole Genome Shotgun project has been deposited at DDBJ/EMBL/GenBank under the accession AXZM00000000. The version described in this paper is version AXZM01000000.

## Electronic supplementary material

Additional file 1: **Heterogeneous sequence (k-mer) and repeat rate analyses of** ***L. rhinocerotis*** **genome.** (PDF 207 KB)

Additional file 2: Table S1: Gene distribution of *L. rhinocerotis* and the other seven compared Basidiomycota fungi in the third layer of KEGG pathway. **Table S2.** Enzyme distribution of *L. rhinocerotis* with not less than 2-fold genes more than the other seven compared Basidiomycota fungi in the fourth layer of KEGG pathway. **Table S3.** The classes of 26 enzymes of *L. rhinocerotis* with not less than 2-fold genes more than the other seven compared Basidiomycota fungi in the fourth layer of KEGG pathway. **Table S4.** The 535 enzymes which are exclusive to only *L. rhinocerotis*. **Table S5.** Cytochrome P450 genes in *L. rhinocerotis*. **Table S6.** The putative backbone genes and clusters involved in *L. rhinocerotis* secondary metabolism. **Table S7.** Putative genes involved in the biosynthesis of bioactive polysaccharides and proteins in *L. rhinocerotis*. **Table S8.** Download portal of additional fungi used in this study. (XLS 298 KB)

Additional file 3: **Assignment of** ***L. rhinocerotis*** **genes to CAZy families and their respective EC activities.** (XLSX 31 KB)

Additional file 4: **Potential terpenoids biosynthesis pathway in** ***L. rhinocerotis*** **.** (PDF 120 KB)
