# Supplementary material for: The genome of the Tiger Milk mushroom, Lignosus rhinocerotis, provides insights into the genetic basis of its medicinal properties
Source: BMC Genomics. 2014 Jul 29;15(1):635. doi: 10.1186/1471-2164-15-635 (PMC4129116; doi:10.1186/1471-2164-15-635)
Supplement: Supplementary file 1 — Additional file 1: Heterogeneous sequence (k-mer) and repeat rate analyses of L. rhinocerotis genome. (PDF 207 KB) [file 12864_2014_6333_MOESM1_ESM.pdf]

Heterogeneous sequence (k-mer) analysis

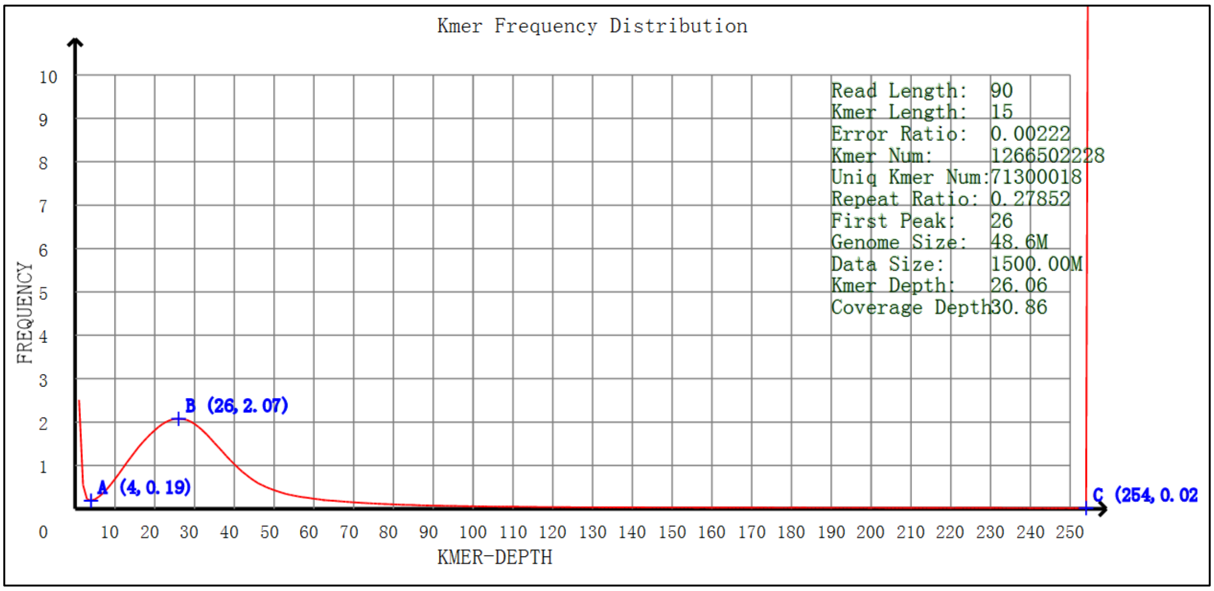

Figure 1 K-mer (15-mer) analysis of *L. rhinocerotis* genome. The genome size of *L. rhinocerotis* is estimated to be 48.6 Mb based on the 15-mer analysis (Liu et al., 2013). The absence of additional peak at ½ of the k-mer depth of the main peak (k-mer depth = 26) suggests that the genome sequences do not exhibit high level of heterozygosity [16]. The k-mer distribution should follow the Poison distribution but low-depth k-mer take up slightly higher proportion in this case. This is most likely due to sequencing error but not heterozygosity. Reads with low sequence quality, significant poly-A structure and kmer frequency of 1 were subsequently removed prior to assembly.

Additional reference:

Liu B, Shi Y, Yuan J, Hu X, Zhang H, Li N, Li Z, Chen Y, Mu D, Fan W: **Estimation of genomic characteristics by analyzing k-mer frequency in *de novo* genome projects.** 2013, arXiv:1308.2012.

Repeat rate analysis

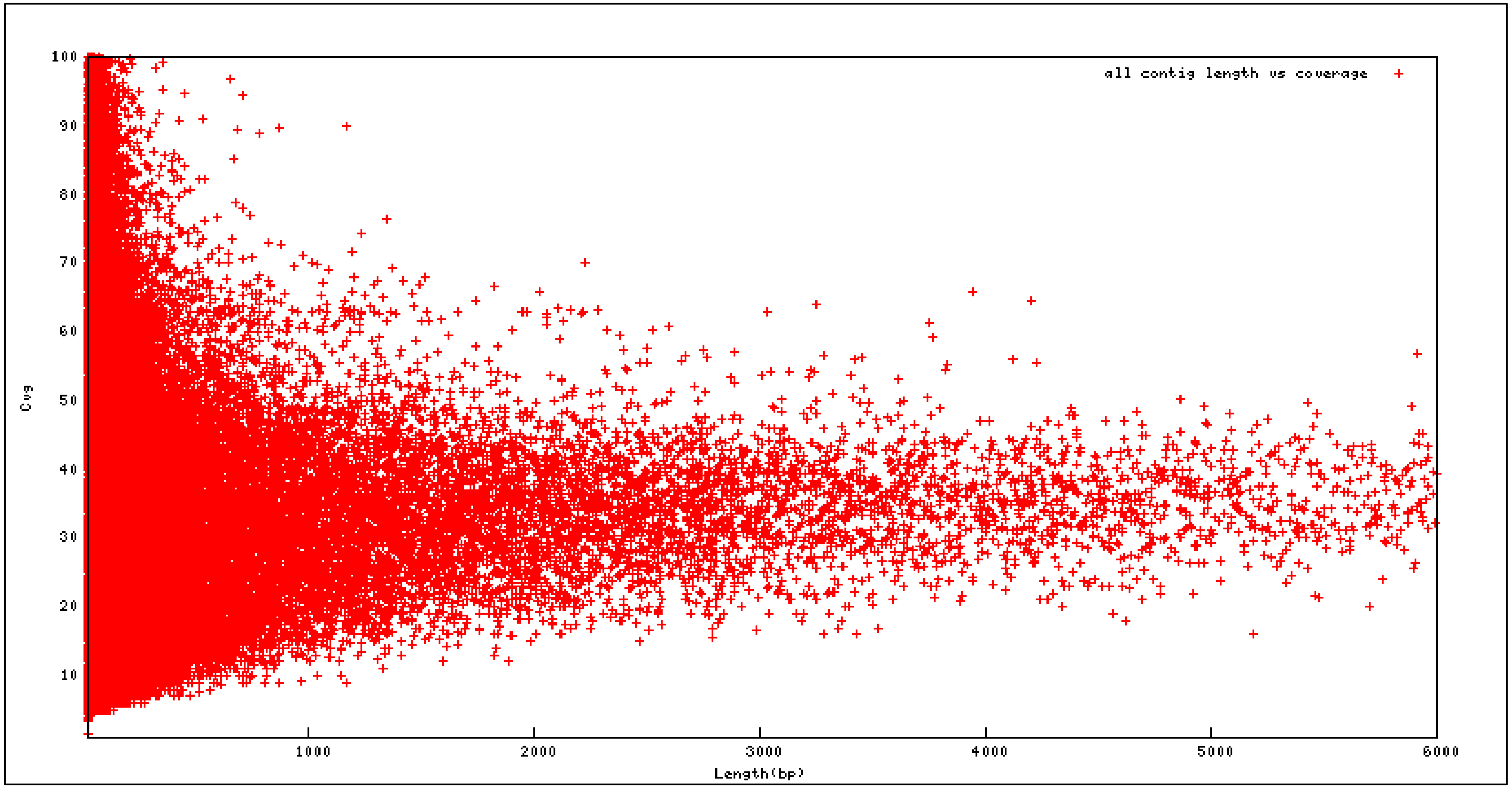

Figure 2 Contig length and coverage distribution. Based on the contig length and depth analysis, contigs with a certain length are selected to calculate their average coverage as the average coverage of the genome.

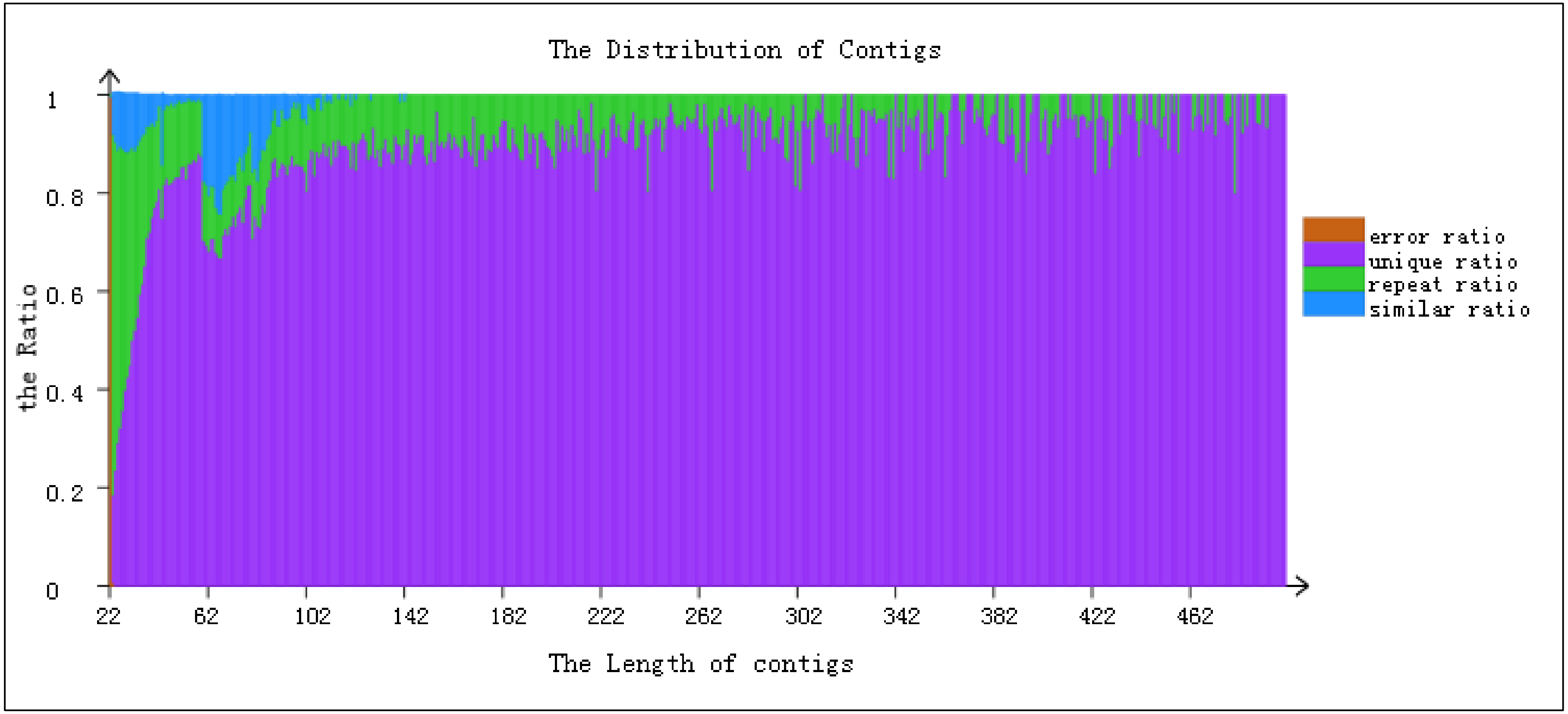

Figure 3 Contig classification graph. The contig classification analysis for different length of contigs contains unique ratio, repeat ratio, similar ratio, and error ratio where error is the average coverage with less than 0.1-fold coverage; repeat is the average coverage with more than 1.8-fold coverage; similar is defined as the similarity higher than 0.95 between contigs of the same length with the average coverage between 0.1-fold and 1.8-fold; and others are unique. The ratio was calculated as shown in Table 1.

Table 1 Repeat rate statistics

	Number of contig	Length (bp)	Number of repeat	Repeat length (bp)	Ratio (%)	Length ratio (%)
**Total contig**	840,965	56,184,612	222,416	7,191,525	26.44	12.79
**Short contig**	805,336	25,402,651	219,603	6,473,589	27.26	25.48
**Long contig**	35,629	30,781,961	2,813	717,936	7.89	2.33

Short contig is contig with the length less than 100 bp and the others are long contig; repeat contig includes the short contig with average coverage no less than 1.5-fold and long contig with average coverage no less than 1.8-fold. The repeat rate of contig with length shorter than 100 bp and longer than 100 bp is 27.26 % and 7.89 %, respectively. Repeat rate of the whole genome is 26.44 %. The total length of repeat fragment is 7,191,525 bp.
