# Supplementary material for: The genome of the Tiger Milk mushroom, Lignosus rhinocerotis, provides insights into the genetic basis of its medicinal properties
Source: BMC Genomics. 2014 Jul 29;15(1):635. doi: 10.1186/1471-2164-15-635 (PMC4129116; doi:10.1186/1471-2164-15-635)
Supplement: Supplementary file 4 — Additional file 4: Potential terpenoids biosynthesis pathway in L. rhinocerotis . (PDF 120 KB) [file 12864_2014_6333_MOESM4_ESM.pdf]

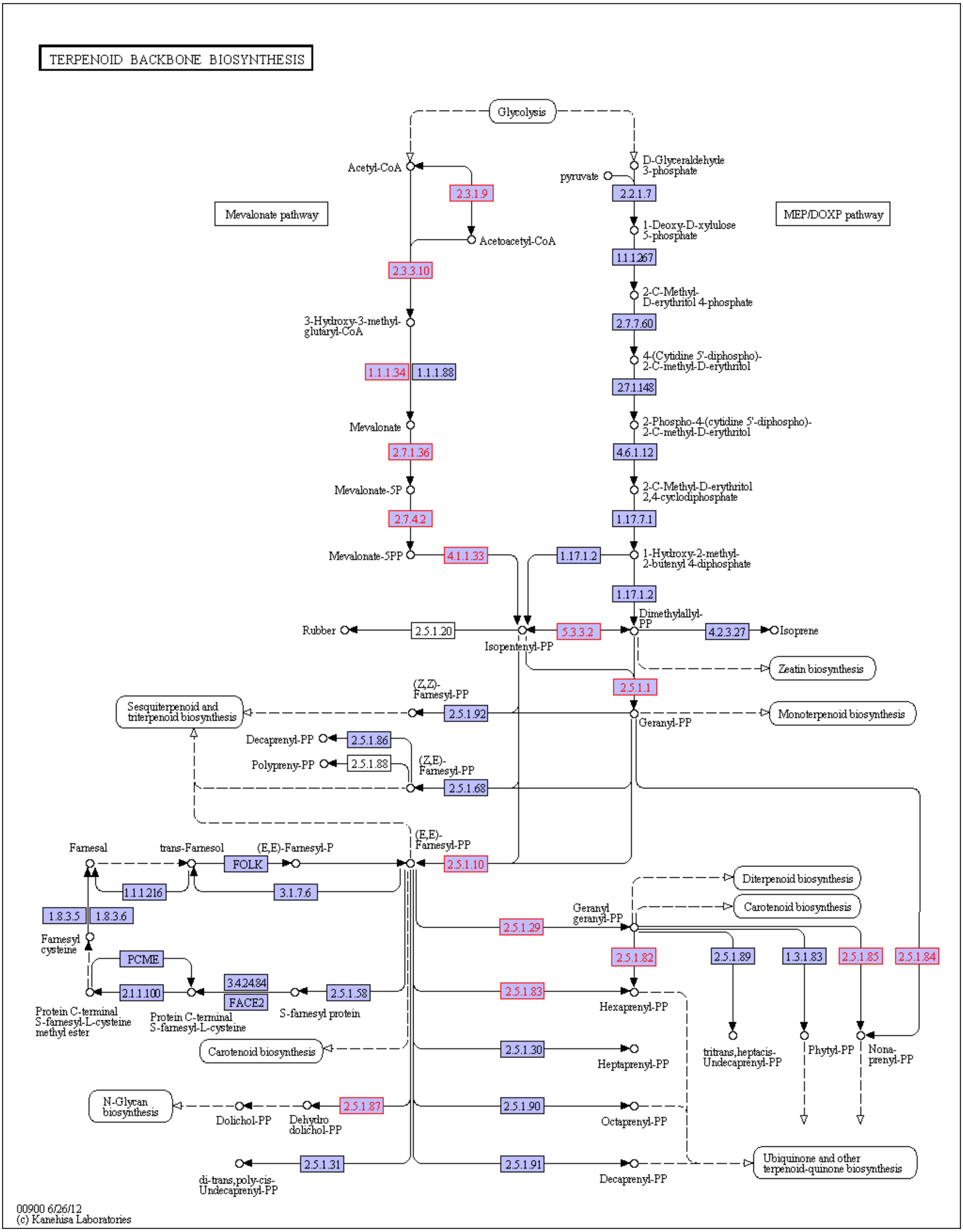

The terpenoid backbone biosynthesis pathway of *L. rhinocerotis* by KEGG mapper (http://www.genome.jp/kegg/mapper.html). Blue box marked in red indicates existing homologous genes of the enzyme.
